# Roles of kininogen-1, basement membrane specific heparan sulfate proteoglycan core protein, and roundabout homolog 4 as potential urinary protein biomarkers in diabetic nephropathy

**DOI:** 10.17179/excli2020-1396

**Published:** 2020-06-24

**Authors:** Piyada Na Nakorn, Supitcha Pannengpetch, Patcharee Isarankura-Na-Ayudhya, Chadinee Thippakorn, Ratana Lawung, Nuankanya Sathirapongsasuti, Chagriya Kitiyakara, Piyamitr Sritara, Prin Vathesatogkit, Chartchalerm Isarankura-Na-Ayudhya

**Affiliations:** 1 Department of Clinical Microbiology and Applied Technology, Faculty of Medical Technology, Mahidol University, Bangkok, Thailand; 2 Center for Research and Innovation, Faculty of Medical Technology, Mahidol University, Nakornpathom, Thailand; 3 Department of Medical Technology, Faculty of Allied Health Sciences, Thammasat University, Pathumthani, Thailand; 4 Section for Translational Medicine, Research Center, Faculty of Medicine, Ramathibodi Hospital, Mahidol University, Bangkok, Thailand; 5 Department of Medicine, Faculty of Medicine, Ramathibodi Hospital, Mahidol University, Bangkok, Thailand

**Keywords:** diabetic nephropathy, urine, quantitative proteomics

## Abstract

Diabetic nephropathy, a major complication of diabetes mellitus (DM), is increasing worldwide and the large majority of patients have type 2 DM. Microalbuminuria has been used as a diagnostic marker of diabetic nephropathy. But owing to its insufficient sensitivity and specificity, other biomarkers are being sought. In addition, the pathophysiology of diabetic nephropathy is not fully understood and declines in renal function occur even without microalbuminuria. In this study, we investigated urinary proteins from three study groups (controls, and type 2 diabetic subjects with or without microalbuminuria). Non-targeted label-free Nano-LC QTOF analysis was conducted to discover underlying mechanisms and protein networks, and targeted label-free Nano-LC QTOF with SWATH was performed to qualify discovered protein candidates. Twenty-eight proteins were identified as candidates and functionally analyzed via String DB, gene ontology and pathway analysis. Four predictive mechanisms were analyzed: i) response to stimulus, ii) platelet activation, signaling and aggregation, iii) ECM-receptor interaction, and iv) angiogenesis. These mechanisms can provoke kidney dysfunction in type 2 diabetic patients via endothelial cell damage and glomerulus structural alteration. Based on these analyses, three proteins (kininogen-1, basement membrane-specific heparan sulfate proteoglycan core protein, and roundabout homolog 4) were proposed for further study as potential biomarkers. Our findings provide insights that may improve methods for both prevention and diagnosis of diabetic nephropathy.

## Introduction

Type 2 diabetes mellitus (T2DM) is a metabolic disorder associated with microvascular complications. Diabetic nephropathy frequently leads to end-stage renal disease (ESRD) with a high-risk of death (Tziomalos and Athyros, 2015[56]). The prevalence of nephropathy in T2DM is approximately 30-50 % overall and higher in patients older than 65 years (Pavkov et al., 2006[42]). The rate of progression to diabetic nephropathy is quite difficult to predict. Microalbuminuria is known as a risk factor for ESRD and death (Berhane et al., 2011[3]). However, it is not an accurate predictor of diabetic nephropathy. Moreover, impaired kidney function in the absence of microalbuminuria occurs (Antic et al., 2009[1]; Gawandi et al., 2018[14]). Therefore, more reliable markers are required for better detection of kidney dysfunction in T2DM. 

Urine provides a desirable biological sample for diagnosis and monitoring of diseases because of its low complexity and non-invasive access (Shoukry et al., 2015[50]; Wang et al., 2014[58]). The protein concentration of urine is lower than that of blood and other biological samples. However, urine contains both plasma-derived and kidney-derived proteins, reflecting the pathological and physiological status of kidney (Pieper et al., 2004[45]). Therefore, urine is suitable for discovery of biomarkers which reflect underlying mechanisms that affect renal or pre-renal processes. Proteomics assesses the entire protein profiles of biological fluids under various conditions, and can provide a technological approach to nephrology through the urinary proteome (Fliser et al., 2007[12]). For decades urinary proteins have been studied in an effort to discover biomarkers of kidney diseases, notably diabetic nephropathy (Papale et al., 2010[40]; Pastushkova et al., 2018[41]; Tam et al., 2009[53]; Yu et al., 2017[60]).

The pathophysiology of diabetic nephropathy remains incompletely understood. Microalbuminuria is the method most commonly used for detecting diabetic nephropathy. Occurrence of microalbuminuria is mainly due to glomerular damage which results in impairment of the glomerular filtration barrier. However, impaired renal function can occur prior to the presence of microalbuminuria (Caramori et al., 2003[5]; Perkins et al., 2007[43]). This suggests that the onset of diabetic nephropathy may be driven by several different mechanisms.

A number of candidate urinary biomarkers of diabetic nephropathy have been identified, and they reflect different potential forms of kidney injury (Lee and Choi, 2015[25]). The lead candidate is urinary transferrin which has been claimed to be more sensitive than microalbuminuria as a predictor of diabetic nephropathy. An increase of transferrin has been found in diabetic patients even without microalbuminuria (Narita et al., 2004[36]; Zhang et al., 2019[61]). However, it is noteworthy that urinary transferrin is not specific to diabetic nephropathy and also increases in other diseases that damage the glomerulus (Mackinnon et al., 2003[31]). 

To discover other potential biomarkers for diabetic nephropathy, we used a mass spectrometry (MS)-based proteomics approach. This technique has been used for decades, and several studies of diabetic nephropathy used quantitative proteomic techniques including both labeling and label-free approaches (Duangkumpha et al., 2019[11]; Overgaard et al., 2010[38]). The label-free approach is frequently used because it is cheaper and provides higher throughput and sensitivity than the labeling approach. The approach may be enhanced with SWATH (sequential window acquisition of all theoretical fragment ion-mass spectrometry), which increases consistency and reproducibility while also providing an elaborate data set (Lin et al., 2018[28]; Muntel et al., 2015[35]).

In this study, we sought underlying mechanisms and potential urinary protein markers using the non-targeted label-free Nano-liquid chromatography quadrupole time of flight (LC QTOF) analysis coupled with a powerful bioinformatics software. In parallel, we used a targeted label-free Nano-LC QTOF analysis via SWATH to assess these candidates as biomarkers for kidney dysfunction in T2DM. 

## Materials and Methods

### Sample collection

This study was conducted in accord with the Helsinki Declaration and approved by the Committee for Research of Faculty of Medicine, Ramathibodi Hospital, Mahidol University, Bangkok, Thailand (Protocol approval ID-05-51-19). Mid-stream urine and data were collected from former employees of EGAT (The Electric Generating Authority of Thailand) who were participants in the EGAT Project, an ongoing prospective cohort in Thailand which assesses metabolic risk for cardiovascular disease and other chronic diseases. All EGAT participants have a health survey every 5 years. Our subjects were grouped as follows: i) controls, ii) those with type 2 diabetes mellitus, and iii) those with type 2 diabetes mellitus with microalbuminuria (T2DM with MAU). The participants who had no history of DM in the EGAT Project study year 2013 record and an 11-pad urine strip test in year 2018 showing all negative pads were considered as controls. Type 2 DM status was verified by a normal blood glucose test: 70-99 mg/dL. The participants who had a history of DM in year 2013's record and an 11-pad urine strip test showing all negative pads with or without a positive glucose pad were considered as having T2DM. The participants who had a history of DM in year 2013's record and 11-pad urine strip test showing all negative pads along with a positive (trace to 3+) protein pad (with or without positive glucose pad) were considered as having T2DM with MAU. Microalbuminuria was defined as a urinary albumin-creatinine ratio between 30 and 300 mg/g. All 3 groups were matched by age range, sex ratio, body mass index, and duration of diabetes mellitus. Exclusion criteria were as follows: diabetes mellitus type 1, uncontrolled hyperglycemia (HbA1c), uncontrolled blood pressure, history of kidney diseases other than diabetic nephropathy, estimated glomerular filtration rate (eGFR) < 60 ml/min/m^2^, urinary tract infection, malignancy, hyperlipidemia, liver disease, and use of angiotensin inhibitors or angiotensin receptor blockers. In summary, 24 subjects were assigned to the control group, 18 to the T2DM group, and 4 to the T2DM with MAU group. Characteristics of the groups, such as age, sex and blood chemical parameters, are listed in Table 1(Tab. 1).

### Sample preparation

Approximately 50 ml of urine was collected from each subject using standard procedures for storage and sample transportation. The samples were centrifuged at 4,000 rpm, for 15 min for cell debris removal. Supernatant was collected and stored at -80 °C until further use. The urine samples were divided into 2 parts, for discovery and biomarker qualification phases. The discovery phase was conducted using pooled samples, while individual samples were used in the biomarker qualification phase. The final volume of all biological replicates of both phases was 3 ml. For discovery phase, urine samples were divided into 4 biological replicates. Then equal volumes of samples were pooled to be used as a single biological replicate. The exception was that T2DM with MAU subjects were each used as biological replicates because there were only four of them. Desalting and precipitation of urinary proteins were conducted using 75 % ethanol coupled with a 2D-clean up kit (GE Healthcare, USA). Precipitated protein was resuspended in lysis buffer (7 M urea, 2 M thiourea, 4 % CHAPS, and 1X protease inhibitor cocktail) with 1X protease inhibitor cocktail, and then cleaned with the 2D-clean up kit. After that, the pellet was resuspended in lysis buffer and protein concentration was determined using a Bradford assay. In the biomarker qualification phase, individual urine samples were used but there was not enough protein in some samples. These samples were pooled and resulted in 17, 17, and 4 individual samples of controls, T2DM, and T2DM with MAU, respectively.

### In-solution digestion

At first, acetone precipitation was conducted in order to buffer changing from lysis buffer to 8 M urea that was more suitable for in-solution digestion. For each sample, 100 µg of protein was reduced by incubation at room temperature for 30 min in reduction buffer (100 mM dithiothreitol in 100 mM TEAB). Then an alkylating buffer (100 mM iodoacetamide in 100 mM TEAB) was added and the sample was incubated at room temperature in the dark for 30 min. It was then incubated again with reduction buffer at room temperature for 15 min for quenching. Ice-cold acetone was added and the sample incubated at -20 °C overnight, then centrifuged to collect the pellet. The protein pellet was resuspended with 8 M urea in 100 mM TEAB and then digested for 16 hours at 37 °C using Trypsin, Gold (mass spectrometry grade; Promega, USA). The sample was dried in a CentriVap DNA Concentrator (Labconco Co., Kansas City, Missouri, USA), resuspended in 0.1 % formic acid (FA), and cleaned up by C18 Zip tip. The cleaned peptide was then dried in the CentriVap and stored at -80 °C until further processing. Finally, the sample was resuspended in 0.1 % formic acid and the peptide concentration measured by Nano drop 1000 (Thermo Fisher Scientific, Bremen, Germany).

### Label-free Nano-LC-MS/MS analysis

#### Non-targeted label-free Nano-LC QTOF for discovery phase

Peptides were analyzed on an LC-MS/MS system including a Nano-liquid chromatograph (Dionex Ultimate 3000, RSLCnano System, Thermo Scientific) in combination with a CaptiveSpray source/Quadrupole ion trap mass spectrometer (Model Q-ToF Compact II, Bruker, Germany). One microgram of peptides was enriched by a Nano trap column 100 µm i.d. × 2 cm, Acclaim PepMap100 C18 5 µm, pore size 100 Å and separated using a PepMap100 C18 3 µm 75 µm × 500 mm LC column. Elution was performed using a linear gradient of 2-95 % Solvent B over 90 min at flow rate of 300 nL/min and a column temperature of 60 °C. There were 2 mobile phases: A) 0.1 % FA in water, and B) 0.08 % FA in 80 % acetonitrile. The loading pump solvent consisted of 0.05 % TFA in 2 % acetonitrile. A gradient of mobile phase B was used as follows: 2 % for 5 min, ramped to 30 % for 60 min, then ramped to 50 % for 10 min, ramped to 70 % for 5 min, and ramped to 95 % for 5 min, then ramped down to 2 % for 1 sec and re-equilibrated for 5 min. Drying gas flow and temperature were 5  L/min and 150 °C, respectively, and nebulizer gas pressure was 0.2 bars. MS acquisition rate was 6 Hz and a positive ionization mode was used with a survey scan mass range of m/z 150-2200. AutoMS^n^ CID fragmentation experiments were performed at low (4 Hz) and high (16 Hz) mass spectral rates for the top 2 most intense precursor ions using 3 sec dynamic exclusion. Sodium formate was used for internal calibration, injected with a string pump.

#### Targeted label-free Nano-LC QTOF for biomarker qualification phase using SWATH

Samples from the same subjects as in the discovery phase were used. The operation was conducted using the same system as in the non-targeted analysis. One microgram of peptides was separated by a PepMap100 C18 3 µm 75 µm × 500 mm LC analytical column after enrichment on a Nano trap column 100 µm i.d. × 2 cm, Acclaim PepMap100 C18 5 µm, pore size 100 Å. Mobile phase A consisted of 0.1 % FA in water (HPLC grade); mobile phase B consisted of 0.08 % FA in 80 % acetonitrile. The loading pump solvent consisted of 0.05 % TFA in 2 % acetonitrile; temperature was 60 °C and the flow rate was 300 nL/min. The gradient of mobile phase B was: 2 % at 0-5 min, 30 % at 65 min, 50 % at 75 min, 70 % at 80 min, 95 % at 85 min, and 2 % at 85.1-90 min. In SWATH mode, we used a targeted MRM method that was pre-set in the system, specifically a quadrupole resolution at 25 Da/mass selection. Using an isolation width of 26 Da (containing 1 Da for the window overlap), a set of 32 overlapping windows was constructed covering a precursor mass range of 150-2200 Da.

### MS data processing and statistical analysis

For the discovery phase, raw MS data were processed with MaxQuant software (version 1.6.2.10) coupled with its built-in search engine, Andromeda, for protein identification. The default setting, with the human database downloaded from www.uniprot.org, was set to 1 as label-free approach. Parameter settings used the defaults except the following. Oxidation of methionine and acetylation of the N-terminus were set as variable modifications whilst carbamidomethyl modification of cysteine was set as a fixed modification. Bruker Q-TOF was selected for instrument type, with peptide tolerance for first and main searches set as 0.5 and 0.25, respectively. Trypsin/P was set for the identification of peptides with a maximum of two missed cleavages. Label-free quantification used an LFQ minimum ratio count of 1. Raw data were blasted against *Homo sapiens* UniProt database. False discovery rate (FDR) was set at 1 % of the protein level. TOF MS/MS match tolerance was set at 0.5 Da with label-free quantification. Match between run option in the software was used for mass and retention time's re-calibrating between runs. LFQ was carried out and imported into Perseus software (version 1.6.8.0) for differential expression statistical analysis. One-way ANOVA was used out to compare T2DM versus controls, T2DM with MAU versus controls, and T2DM with MAU versus T2DM, with post-hoc Tukey's HSD test cut-off set at FDR 0.05. Venny (version 2.1) was used for plotting Venn diagrams. In order to conduct multivariate analysis, principal component analysis was performed using R software. Significant differentially expressed proteins were subjected to protein-protein interactions, GO-enrichment, and pathway analysis using String DB (www.string-db.org).

For the biomarker qualification phase, Skyline software (version 19.1.0.193) was used to analyze the data. Twenty-eight proteins, identified as significant in the discovery phase, were analyzed to determine the extent of protein expression. All peaks were checked manually for correct integration and peptide intensity was clarified as the peak area based on the sum of all transitions. The Total Area Sum (TAS) approach was processed manually to determine peptide intensity. Data were visualized by the R software and GraphPad Prism (version 5.01). *P*-values < 0.05 with post-hoc Tukey's HSD test were considered statistically significant. 

## Results

### Clinical characteristics of subjects and experimental workflow in both discovery and biomarker qualification phases

**Figure 1:**
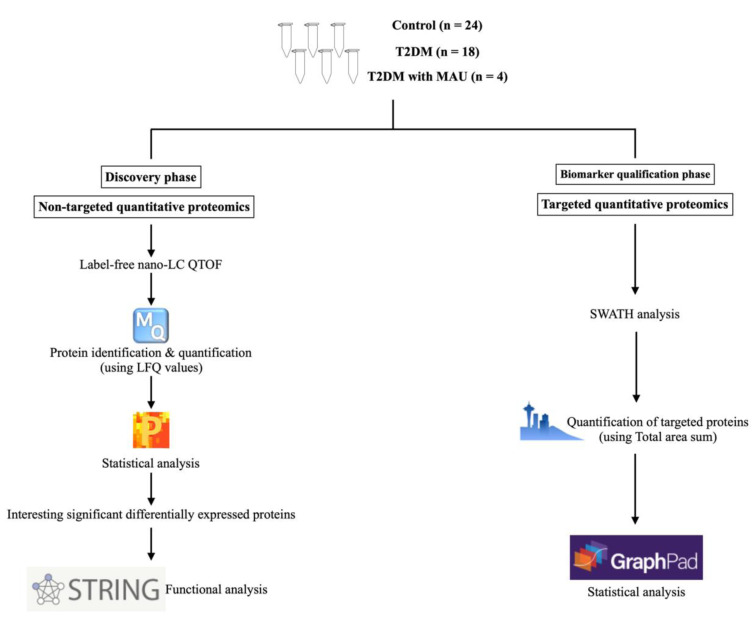
The experimental design for discovery of underlying mechanisms and qualification of urinary biomarkers of diabetic nephropathy. Forty-six urine samples were collected and used in the two phases. The discovery phase was performed using label-free Nano-LC QTOF by a non-targeted quantitative proteomics approach. Protein identification and quantitation were completed by the MaxQuant software, followed by statistical analysis with Perseus software. Functional analysis was done by the String DB. Urinary biomarker qualification for targeted proteins was achieved by the SWATH-MS analysis. Skyline software was used for data processing and GraphPad for statistical analysis.

Experimental design and the tools/software used in this study are presented in Figure 1(Fig. 1). Urine samples were used in two experimental phases, discovery and biomarker qualification. Clinical and laboratory data of the 46 subjects are summarized in Table 1(Tab. 1). There were no significant differences in these characteristics among the 3 groups except for the urinary albumin/creatinine ratios. The medications of subjects in the T2DM and T2DM with MAU groups were similar and so the influence of medicines on the experiment was avoided. Sex ratio, age, and BMI were matched between the 3 groups as follows. For sex ratio, the number of females was less than that of males in all groups. Mean ages of the 3 groups were 61.5±3.6 years in the control group, 62.8±5.6 years in the T2DM group, and 61.3±7.4 years in the T2DM with MAU group. BMI of all 3 groups were similar, 23.3±2.3 kg/m^2^ in the control group, 26±4.3 kg/m^2 ^in the T2DM group, and slightly increased to 32.6±7.5 kg/m^2 ^in the T2DM with MAU. T2DM and T2DM with MAU groups did not significantly differ in mean duration of diabetes (10.9±9.6 and 7.8±5.5 years, respectively). There were also no significant differences among groups regarding blood pressure, nor lipid and liver profiles. Fasting blood glucose was slightly increased in the T2DM and T2DM with MAU groups (122.2±25.6 and 134.3±23.3 mg/dL, respectively) compared to that in the control group (90.5±10.7 mg/dL), with a similar trend in HbA1c results. Urinary albumin/creatinine ratios were higher in T2DM with MAU group than in the control and T2DM groups (which were not significantly different). 

### Discovery phase using non-targeted label-free Nano-LC QTOF

**Figure 2:**
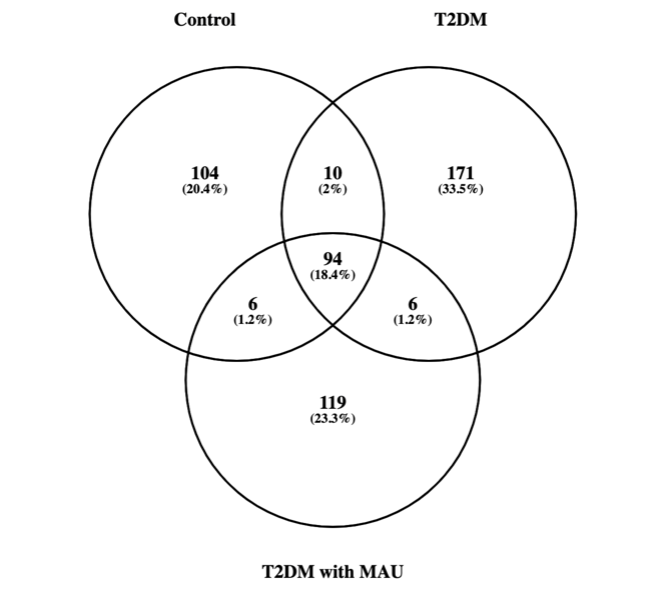
Venn diagram representing the identified proteins using label-free Nano-LC QTOF analysis. There are a total of 510 proteins in 3 sample groups, including controls, T2DM, and T2DM with MAU. Overlapping areas indicated shared proteins between 2 or 3 groups.

Urine proteins from 12 pooled samples [4 biological replicates from the 3 groups: controls (n = 24), T2DM (n = 18), and T2DM with MAU (n = 4)] were compared using label-free Nano-LC QTOF analysis. To achieve the analysis, a technical triplicate analysis of the same pooled sample was conducted on urine sample. Raw data from mass spectrometry, analyzed using MaxQuant software with a 1 % false discovery rate, identified proteins and LFQ values for protein quantification. Of the 510 identified proteins, 214, 281, 225 were in the control, T2DM and T2DM with MAU groups, respectively. From the Venn diagram (Figure 2(Fig. 2)), the 3 groups shared 94 proteins while T2DM had the most unique proteins of 171 proteins. 

**Figure 3:**
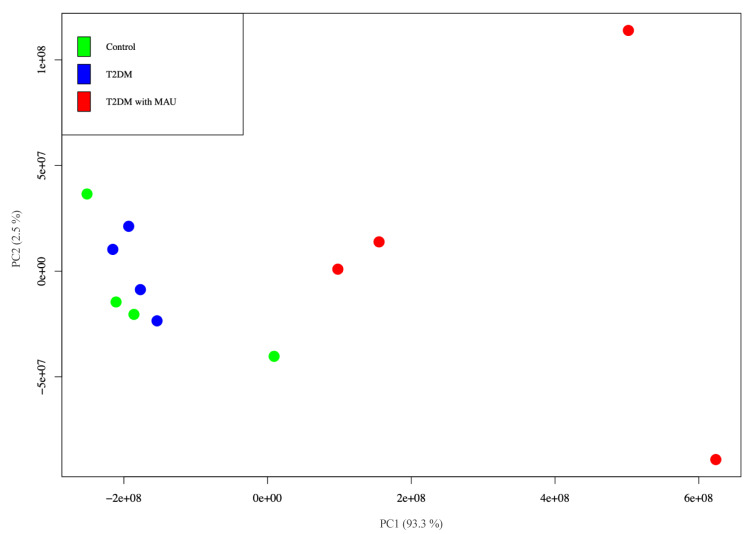
Principal component analysis (PCA) of the LFQ values obtained from urine samples of control (green), T2DM (blue) and T2DM with MAU (red) subjects. Calculations and visualization were performed with R software.

Non-supervised analysis was performed to investigate the capability to differentiate urinary protein profiles. Principal component analysis (PCA) differentiated the T2DM with MAU group from the other 2 groups whilst the analysis revealed similarity of the control and T2DM groups (Figure 3(Fig. 3)). Each spot in the figure represents 1 biological replicate. There is greater reproducibility in control and T2DM groups since the spot distances are relatively close together compared to those of the T2DM with MAU group. The small sample size of this group (n = 4), in which each biological replicate was from 1 sample, resulted in more variability than in the other 2 groups. Using the PCA results, protein expression was done using LFQ values and groups compared using Perseus software. The protein expression ratios were converted to Log2 fold changes. Ratio greater or less than 1 were considered for comparison.

**Figure 4:**
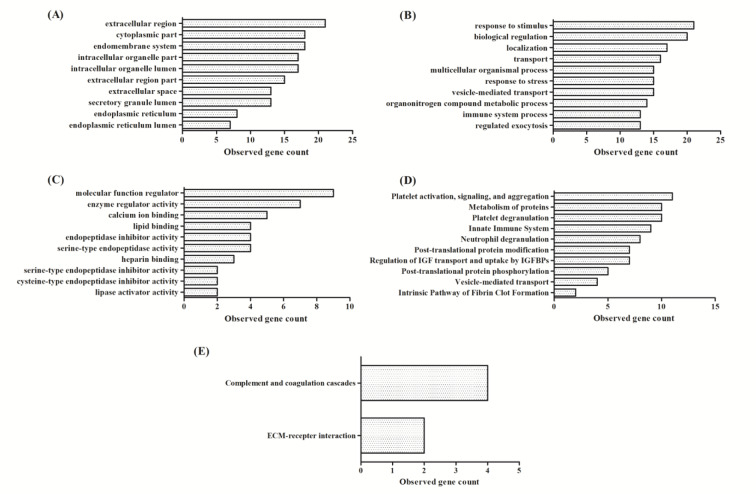
Enrichment of 28 significant differentially-expressed proteins using GO, KEGG, and Reactome pathways. (A) Top 10 enriched cellular components, (B) Top 10 enriched biological processes, (C) Top 10 enriched molecular functions, (D) Top 10 enriched Reactome pathways, and (E) enriched KEGG pathways

One-way ANOVA together with post-hoc Tukey's test revealed 28 significant proteins. We used String DB to investigate protein-protein interactions and explored enrichment of these expressed proteins. Data were classified based on their GO term, molecular function, biological processes and cellular components, KEGG and Reactome pathways. Our analysis showed that 23 of 28 proteins were matched in the String database, the exceptions being the immunoglobulin proteins/fragments [anti-Streptococcal/anti-myosin immunoglobulin kappa light chain variable region (fragment), Ig kappa chain C region, Ig kappa chain V-I region AU, Ig kappa chain V-I region AG, IGK@ protein, MS-F1 light chain variable region (fragment)]. Twenty of 23 proteins had good protein-protein interactions; exceptions were roundabout homolog 4, lithostathine-1-alpha, and prostatin. The majority of these significant urine proteins were extracellular, functioned as a molecular function regulator, and/or involved biological responsiveness to stimulus. The major pathway involved was coagulation as indicated from KEGG and Reactome database results (Figure 4(Fig. 4)). Based on comparison between the control and T2DM with MAU groups, the significant proteins were down-regulated except for alpha-1-acid glycoprotein 1, alpha-1-antichymotrypsin, alpha-1-antitrypsin, alpha-1B-glycoprotein, serotransferrin, and albumin (Table 2(Tab. 2)).

Eleven of 21 proteins involved in platelet activation (derived from Reactome database), while alpha-1-antitrypsin, kininogen-1, mannan-binding lectin serine protease 2, and prothrombin functioned in the complement and coagulation cascades (from KEGG database). Interestingly, all significant proteins were down-regulated (except for serotransferrin and albumin) in the T2DM group compared with the T2DM with MAU group. The Reactome database analysis identified two major pathways, specifically coagulation cascades and immune responses. Moreover, the i) collagen alpha-1 (VI) chain and ii) basement membrane-specific heparan sulfate proteoglycan core protein which functions in ECM-receptor interaction were identified in the analysis of the KEGG database. 

### Biomarker qualification phase using targeted label-free Nano-LC QTOF

**Figure 5:**
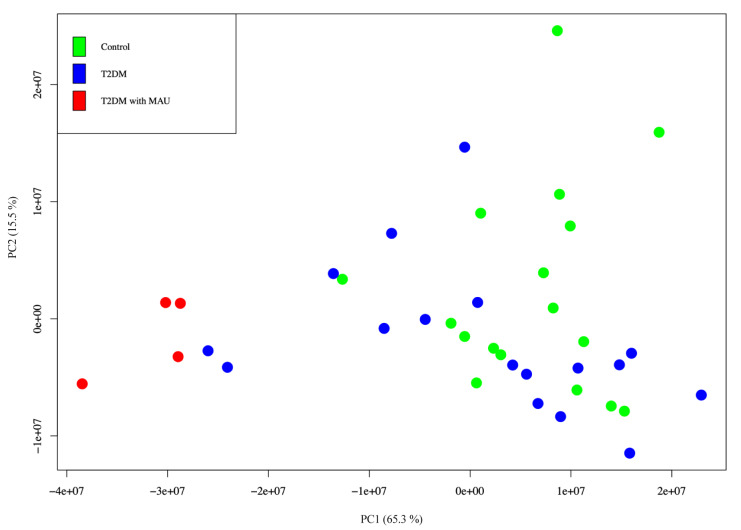
Principal component analysis of urinary proteins from 17 control subjects (green), 17 T2DM subjects (blue), and 4 T2DM with MAU subjects (red). Normalized TAS was calculated and visualized using R software.

Targeted label-free Nano-LC QTOF analysis was used for validation of the 28 significant proteins identified in the discovery phase. Urine samples were used from the same subjects as in discovery phase. Samples with inadequate amounts of protein were combined into single samples. Therefore, there were 17, 17 and 4 subjects in the controls, T2DM, T2DM with MAU groups, respectively. Raw data from mass spectrometry was imported into the Skyline software. Normalized Total Area Sums (Normalized-TASs) were used for comparisons of protein expression. PCA was conducted using R software. Results were in agreement with those found in the discovery phase, that is the T2DM with MAU group was distinct from the control and the T2DM groups (Figure 5(Fig. 5)). There was no obvious clustering among the control and the T2DM groups. However, there were two T2DM subjects that were closely associated with the controls. This was possibly due to biological variation.

**Figure 6:**
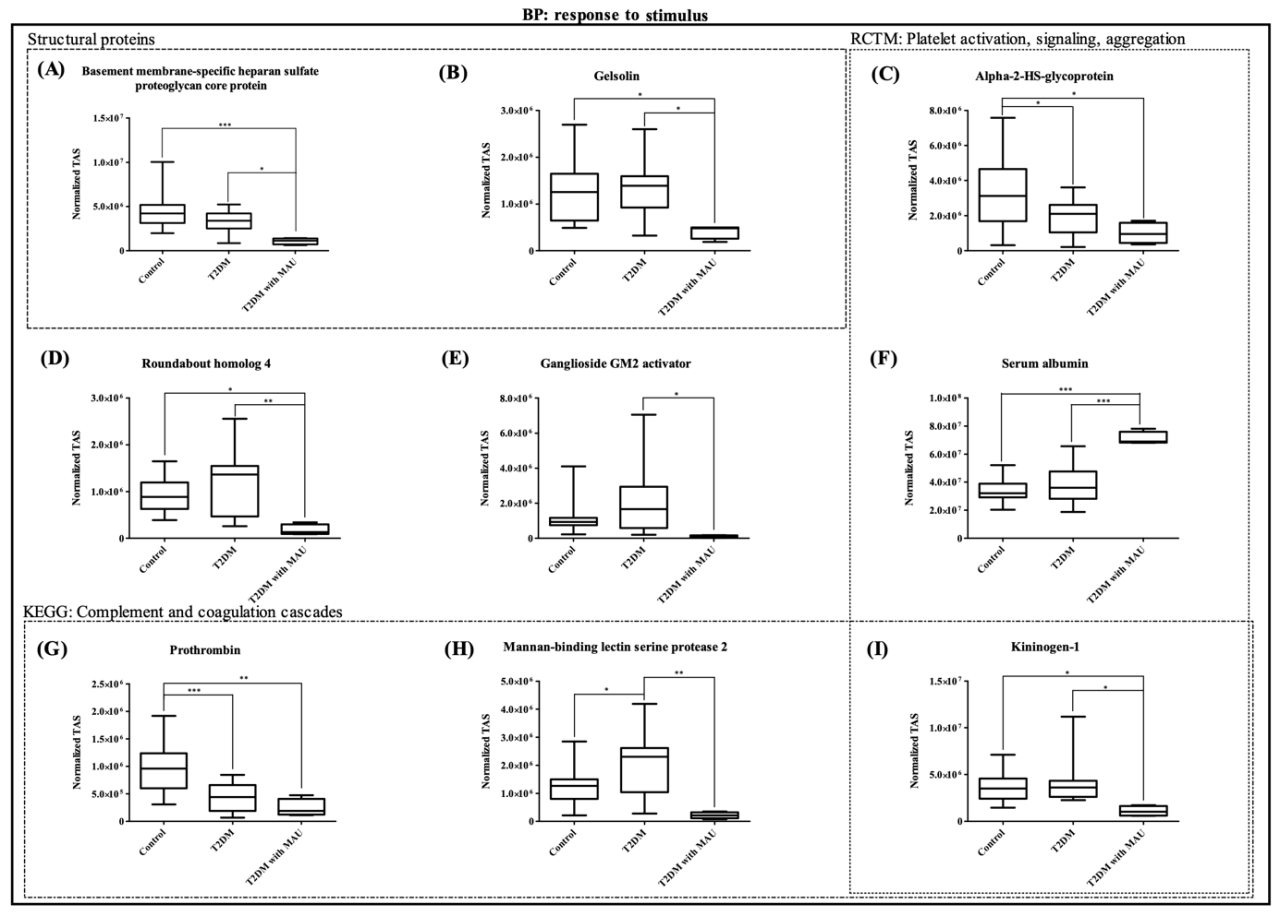
Box plots of 9 significant differentially-expressed proteins in targeted label-free Nano-LC QTOF analyses. All of the proteins were part of the 'in response to stimulus' process. They are divided into 4 subgroups: i) structural proteins, ii) proteins involved in platelet activation, signaling, and aggregation, iii) proteins involved in complement and coagulation cascades, and iv) other proteins. Pairwise group comparisons were calculated using Tukey's multiple comparison test. (* P<0.05, ** P<0.01, *** P<0.001)

Next, the candidate proteins were assessed, using pairwise comparisons, as potential biomarkers. The proteins showed the expression in accordance to the discovery phase, and had ratios of ≥ 1 or ≤ 1, were considered as potential biomarkers of kidney dysfunction in type 2 diabetic patients (Table 2(Tab. 2)). Surprisingly, there were 9 significant differentially-expressed proteins, including albumin which is currently used as a biomarker for diabetic nephropathy. All of the proteins were from the 'response to stimulus' process. Prothrombin and mannan-binding lectin serine protease 2, which are involved in complement and coagulation cascades, were detected. Alpha-2-HS glycoprotein and albumin were found to be expressed during platelet activation, signaling and aggregation. Interestingly, kininogen-1 was found to be involved not only in platelet activation, signaling and aggregation, but also in the complement cascade. Moreover, structural proteins (basement membrane-specific heparan sulfate proteoglycan core protein and Gelsolin) were observed. Figure 6(Fig. 6) presents the results, using targeted label-free Nano-LC QTOF analysis, for: basement membrane-specific heparan sulfate proteoglycan core protein, gelsolin, alpha-2-HS-glycoprotein, roundabout homolog 4, ganglioside GM2 activator, albumin, prothrombin, mannan-binding lectin serine protease 2, and kininogen-1. These box plots show the same trends as the results from the analysis of pooled samples. Alpha-2-HS-glycoprotein and prothrombin were found to be decreased in the disease groups. On the other hand, roundabout homolog 4, ganglioside GM2 activator, and mannan-binding lectin serine protease 2 were found to be increased in the T2DM group and significantly decreased in the T2DM with MAU group. The proteins that were unchanged in the T2DM group but significantly decreased in the T2DM with MAU group were the basement membrane-specific heparan sulfate proteoglycan core protein, gelsolin, and kininogen-1.

## Discussion

Predictive mechanisms implicated in progressive kidney dysfunction in T2DM and qualification of potential urinary biomarkers were investigated in this study. Urine from 3 groups of subjects who were participants in the EGAT project was collected, precipitated, and in-solution digested prior to resolving by label-free Nano-LC QTOF. The label-free quantification method was selected because of its uncomplicated sample preparation and convenience. Each sample was analyzed in triplicates to confirm reproducibility (Onile et al., 2017[37]; Rawat et al., 2016[46]). The Nano-LC QTOF provided a label-free method with a sensitive and precise quantitative approach. In the discovery phase, analyzing pooled samples, the label-free Nano-LC QTOF was performed in combination with MaxQuant software. The method was capable of identifying 510 urinary proteins with more than 2 unique peptides that fell within the range of human urinary proteins, comparable to other proteomics approaches (Duangkumpha et al., 2019[11]; Hirao et al., 2018[18]; Marimuthu et al., 2011[32]). PCA analysis with R software provided explicit separation of the T2DM with MAU group from control and T2DM groups by a shift along the first component. This indicated a difference in protein expression correlated with progressive kidney dysfunction. In contrast, the control and T2DM groups were not distinct, suggesting a similarity of features/proteins that did not change during the progression from non-diabetes to diabetes. However, a study in 2015 reported marked differences among control, T2DM with no nephropathy, and T2DM with nephropathy groups (Heeg et al., 1987[17]; Lewandowicz et al., 2015[26]). Most of the subjects in the T2DM with no nephropathy group took ACEI/ARB, anti-hypertensive drugs, capable of reducing proteinuria. Therefore, hypertensive DM patients taking these drugs may have normal albuminuria even if they have proteinuria. In contrast, our study excluded subjects taking ACEI/ARB drugs from all groups to ensure albuminuria was unaltered. Out of the 510 discovered urinary proteins, 28 proteins were differentially expressed by subjects in the T2DM with MAU group compared with those in the control and T2DM groups. Some of them were previously described as candidate biomarkers for diabetic nephropathy including alpha-1-acid glycoprotein, alpha-1-antitrypsin, collagen fragment, transferrin, uromodulin and albumin (a well-known biomarker) (Cohen-Bucay et al., 2012[8]; Currie and Delles, 2016[9]; Jin et al., 2012[23]). Our 'significant proteins' underwent further functional/pathway analysis. This showed that most were extracellular, related to kidney dysfunction, and involved in response to stimulus, complement cascades, platelet activation, signaling and aggregation, or ECM-receptor interaction mechanisms. 

**Figure 7:**
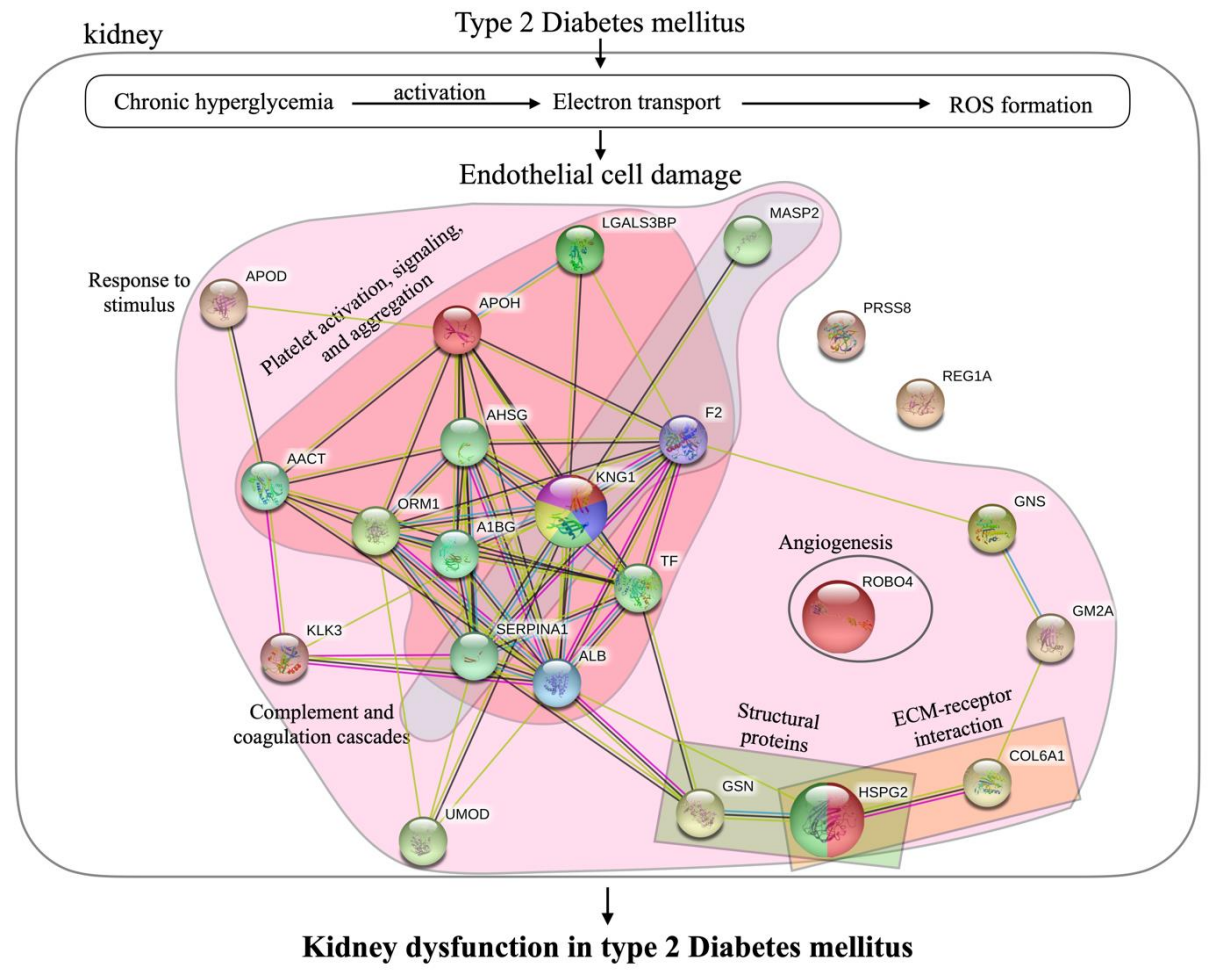
Mechanisms underlying the progressive kidney dysfunction in type 2 diabetes mellitus and responsible proteins. Proteins in pink area are part of the response to stimulus process. Proteins in red and blue areas are involved in platelet activation, signaling and aggregation, and complement and coagulation cascades, respectively. Green and orange areas indicate the proteins that act as structural proteins and serve in ECM-receptor interactions. Larger nodes represent proteins that were chosen to be candidate biomarkers for diabetic nephropathy.

Diabetic nephropathy is a serious complication of diabetes mellitus and may lead to end-stage renal disease (Hovind et al., 2001[19]). The complication progresses gradually to dysfunction of the kidney promoted by various accelerators (Forbes and Fotheringham, 2017[13]; Jefferson et al., 2008[22]; Sagoo and Gnudi, 2018[49]). Chronic hyperglycemia induces microvascular damage by activation of electron transport and provokes ROS formation (Palm et al., 2003[39]; Singh et al., 2008[51]). Particularly in the kidney, accumulation of ROS causes endothelial cell damage. Four predictive mechanisms which lead to progressive diabetic nephropathy by enhancing endothelial cell damage and glomerular alteration are schematically presented in Figure 7(Fig. 7) and summarized as follows.

First, in our T2DM with MAU group, most of the significant proteins were decreased more than two folds compared to those of the control and T2DM groups, and were found to be involved in the response to stimulus process. These findings held for apolipoprotein D, ganglioside GM2 activator, mannan-binding lectin serine protease 2, prothrombin, roundabout homolog 4, and uromodulin. Importantly, 3 of them (kininogen-1, mannan-binding lectin serine protease 2, and prothrombin) involved in the complement cascade, indicating dysfunction of immune response. There are an increasing number of studies which suggest that inflammation is one of underlying processes in diabetic nephropathy, associated with up-regulation of various inflammatory parameters (Donate-Correa et al., 2020[10]; Katsuki et al., 1998[24]; Pickup et al., 2000[44]). However, some inflammatory parameters may be decreased in diabetic nephropathy or increases can be reversed by the effective protective approaches (Sabapathy et al., 2019[47]).

Second, 12 of the significant proteins, e.g. alpha-1-antitrypsin, kininogen-1 and prothrombin, are involved in the coagulation cascade, especially in activation, signaling, and aggregation (both up- and down-regulation). Other studies have shown enhanced coagulation function in diabetic patients with nephropathy (Sun and Liu, 2018[52]). The down-regulation of signaling proteins may drive the reduction of proteins that function in stimulus-response. Interestingly, kininogen-1 was at the core of the aforementioned traffic link. Even though kininogen-1 plays a role in the kallikrein-kinin system (cooperation with renin-angiotensin system), the mechanism remains unclear. In type 1 DM with MAU, both plasma and urinary kininogen-1 have been proposed as predictors of disease (Merchant et al., 2013[33]; Tomita et al., 2012[55]; Vitova et al., 2017[57]).

Third, glomerulus structural alteration is mentioned frequently in the pathophysiology of the kidney dysfunction of DM patients. In the current study, we found expressed proteins which were implicated in ECM-receptor interactions and acted as structural proteins. Thickening of the glomerular basement membrane (GMB) is a structural change found in diabetic nephropathy. Collagen alpha-1(IV) chain and basement membrane-specific heparan sulfate proteoglycan core protein are glomerular matrix proteins. Our results agree with reports that these proteins are decreased in diabetic nephropathy (Ikeda et al., 1991[21]; Merchant et al., 2009[34]; Yagame et al., 1995[59]; Zhu et al., 1994[62]). A reduction of extracellular matrix excretion may explain the accumulation of extracellular matrix. Another structural protein we identified was gelsolin that plays a crucial role in regulation of actin filament assembly and disassembly. An actin-based contractile system is used by podocytes to form the specialized epithelial cell covering of the outer layer of glomerular basement membranes (He et al., 2013[16]). Therefore, the reduction of gelsolin found in our study may be responsible for the glomerular alteration in diabetic nephropathy. A decrease of gelsolin in plasma may be involved in ROS production and inflammation which promotes PKC activation and causes kidney damage in diabetic nephropathy (Cheng et al., 2017[7]; Li et al., 2009[27]; Lu et al., 2019[29]; Sagawa et al., 2003[48]). 

Fourth, glomerulus structural alteration progresses because of microvascular impairment caused by endothelial cell damage. This is proposed as playing a crucial role in diabetic nephropathy (Cheng and Harris, 2014[6]; Tervaert et al., 2010[54]). Roundabout homolog 4 (ROBO4) is a member of the Robo protein family, thought to be expressed mainly in the neuronal system. But it is now seen as specific to endothelial cells and to function in the control of angiogenesis (Cai et al., 2015[4]; Huminiecki, 2019[20]). Vascular dysfunction is reported in association with Robo1 as shown in an miRNA study. However, an *in vivo* study found that ROBO4 is crucial for coordinating the symmetry of intersomitic vessels (Bedell et al., 2005[2]; Hartmann and Thum, 2011[15]). Interestingly, our study found that decreased expression of ROBO4 in the T2DM with MAU group was strongly associated with endothelial cells. Therefore, ROBO4 may be responsible for the dysfunction of angiogenesis, causing lesions in glomeruli and lead to kidney dysfunction in diabetic patients. 

In the biomarker qualification phase, we performed SWATH and used a label-free Nano-LC QTOF analysis. Sequential window acquisition of all theoretical mass spectra (SWATH-MS) was performed in MRM mode of the QTOF analysis. In this mode, all ionized peptides within a specified mass range were fragmented using slightly overlapping precursor isolation windows with 25 *m/z* each in a systematic analysis. Though data analysis of SWATH-MS is the hardest among the methods of quantitative MS analysis, it provides comprehensive detection of peptides and has high consistency (Ludwig et al., 2018[30]). The 28 proteins found to be significant from the discovery phase were targeted and their expression was assessed in individual samples. Finally, 9 proteins showed expression levels similar to the results of the discovery phase and reached statistical significance.

For decades, many researchers have tried to use a biomarker to predict the onset of kidney dysfunction in diabetic patients, but with little success. There has been low consistency across studies and validation problems with individual samples (Cohen-Bucay and Viswanathan, 2012[8]). The kidney dysfunction in type 2 DM appears to progress by co-occurrence of multiple mechanisms including inflammation, coagulation, glomerular structural alteration, and angiogenesis. Therefore, we selected proteins showing differential expression and involvement in the proposed mechanisms as candidate biomarkers for diabetic nephropathy in type 2 DM. These were kininogen-1, basement membrane-specific heparan sulfate proteoglycan core protein, and roundabout homolog 4. However, the study was limited by the small number of subjects in the T2DM with MAU group (only 4 subjects). Biological diversity was detected and is seen in the PCA results. Therefore, while this study gained insights into candidate biomarkers for kidney dysfunction in type 2 DM, further quantitative studies with larger numbers of subjects are needed to reliably determine their sensitivity and specificity.

## Conclusion

Diabetic nephropathy is one of the serious complications in diabetes mellitus patients. With the use of such high throughput technology as label-free Nano-LC QTOF integrated with powerful software, the underlying mechanisms of kidney dysfunction in T2DM were shown to be i) dysfunction of the response to stimulus process, ii) platelet activation, signaling, and aggregation, iii) malfunction of ECM-receptor interactions, and iv) angiogenesis. Via these mechanisms endothelial cells are damaged, resulting in glomerulus structural alteration. SWATH analysis, a comprehensive and precise method, provided three candidate biomarkers for kidney dysfunction in T2DM: kininogen-1, basement membrane-specific heparan sulfate proteoglycan core protein, and roundabout homolog 4. After further evaluation, these biomarkers may become effective tools, replacing or in combination with microalbuminuria, for better diagnosis of diabetic nephropathy.

## Conflict of interest

The authors declare that they have no conflict of interest. The funders had no role in the design of the study; in the collection, analyses, or interpretation of data; in the writing of the manuscript, nor in the decision to publish the results. 

## Acknowledgements

This research project was supported by Mahidol University and funded by the Young Researcher Development Program from the National Research Council of Thailand. The authors would like to thank Ramathibodi Hospital, Mahidol University, for assistance in clinical examinations and data entry. We thank the staff members of the EGAT for participating in this study. We would like to thank Ms. Nisakorn Thongmung (Office of Research Academic and Innovation, Biochemistry and Chemical Analysis Unit, Faculty of Medicine, Ramathibodi Hospital, Mahidol University) for her wholehearted and efficient collaboration, Miss Jeeraporn Sawasdikul and Miss Lalida Kawsakul (Section for Translational Medicine, Research Center, Faculty of Medicine, Ramathibodi Hospital, Mahidol University) for their dedicated help in EGAT sample collection. We also thank to Mrs. Poorichaya Somparn (Research Affairs, Faculty of Medicine, Chulalongkorn University) for her assistance with MaxQuant analysis and Dr. Prasong Khaenam (Center for Standardization and Product Validation, Faculty of Medical Technology, Mahidol University) for his kind assistance with the R program.

## Ethical statement

This study was approved by the Committee for Research of the Faculty of Medicine, Ramathibodi Hospital, Mahidol University, Bangkok, Thailand (Protocol approval ID-05-51-19).

## Figures and Tables

**Table 1:**
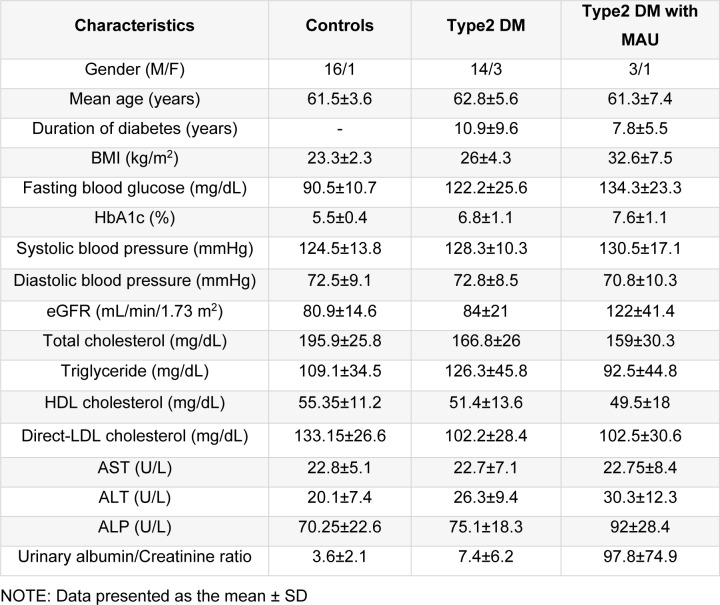
Clinical and laboratory information of controls, type 2 diabetes mellitus (T2DM) and type 2 diabetes mellitus with microalbuminuria (T2DM with MAU) groups

**Table 2:**
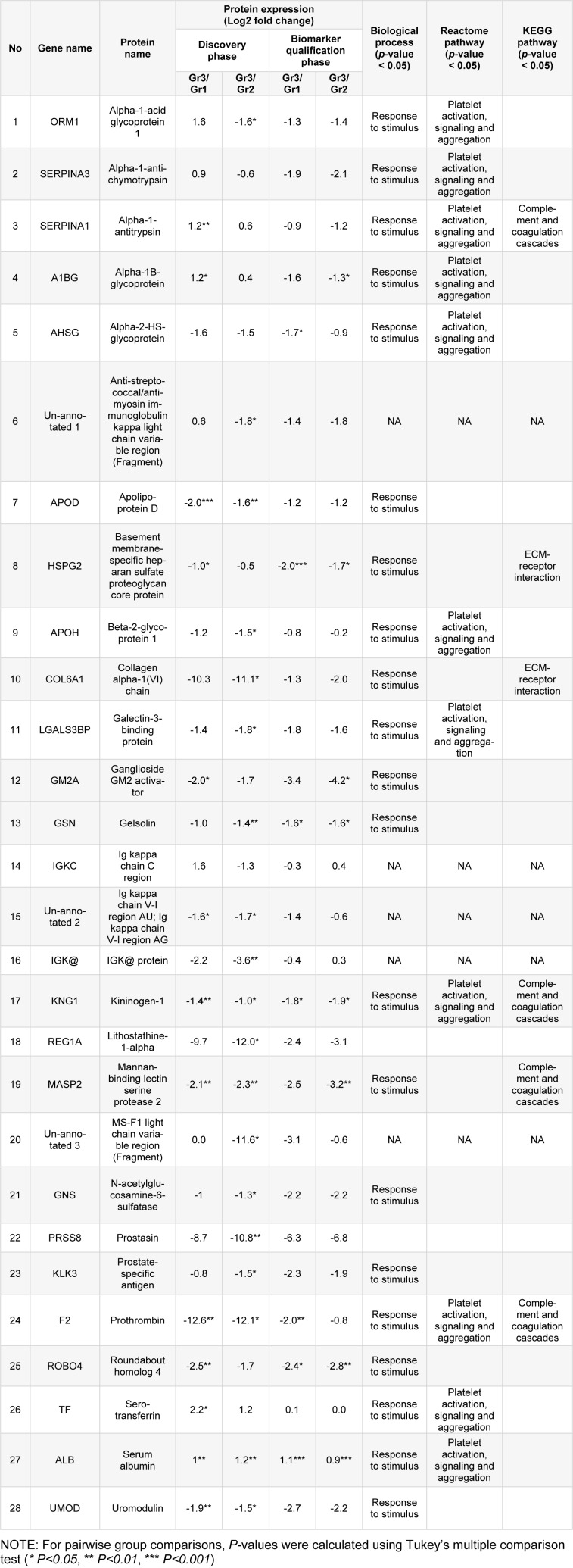
Comparison among groups of expression of proteins identified in the discovery and biomarker qualification phases of study

## References

[1] Antic M, Jotic A, Radovic M, Seferovic JP, Lalic NM, Jovanovic D (2009). Srp Arh Celok Lek.

[2] Bedell VM, Yeo SY, Park KW, Chung J, Seth P, Shivalingappa V (2005). Roundabout4 is essential for angiogenesis in vivo. Proc Natl Acad Sci U S A.

[3] Berhane AM, Weil EJ, Knowler WC, Nelson RG, Hanson RL (2011). Albuminuria and estimated glomerular filtration rate as predictors of diabetic end-stage renal disease and death. Clin J Am Soc Nephrol.

[4] Cai H, Liu W, Xue Y, Shang X, Liu J, Li Z (2015). Roundabout 4 regulates blood–tumor barrier permeability through the modulation of ZO-1, occludin, and claudin-5 expression. J Neuropath Exp Neur.

[5] Caramori ML, Fioretto P, Mauer M (2003). Low glomerular filtration rate in normoalbuminuric type 1 diabetic patients: an indicator of more advanced glomerular lesions. Diabetes.

[6] Cheng H, Harris RC (2014). Renal endothelial dysfunction in diabetic nephropathy. Cardiovasc Hematol Disord Drug Targets.

[7] Cheng Y, Hu X, Liu C, Chen M, Wang J, Wang M (2017). Gelsolin inhibits the inflammatory process induced by LPS. Cell Physiol Biochem.

[8] Cohen-Bucay A, Viswanathan G (2012). Urinary markers of glomerular injury in diabetic nephropathy. Int J Nephrol.

[9] Currie G, Delles C (2016). Urinary proteomics for diagnosis and monitoring of diabetic nephropathy. Curr Diab Rep.

[10] Donate-Correa J, Luis-Rodriguez D, Martin-Nunez E, Tagua VG, Hernandez-Carballo C, Ferri C (2020). Inflammatory targets in diabetic nephropathy. J Clin Med.

[11] Duangkumpha K, Stoll T, Phetcharaburanin J, Yongvanit P, Thanan R, Techasen A (2019). Urine proteomics study reveals potential biomarkers for the differential diagnosis of cholangiocarcinoma and periductal fibrosis. PLoS One.

[12] Fliser D, Novak J, Thongboonkerd V, Argiles A, Jankowski V, Girolami MA (2007). Advances in urinary proteome analysis and biomarker discovery. J Am Soc Nephrol.

[13] Forbes JM, Fotheringham AK (2017). Vascular complications in diabetes: old messages, new thoughts. Diabetologia.

[14] Gawandi S, Gangawane S, Chakrabarti A, Kedare S, Bantwal K, Wadhe V (2018). A study of microalbuminuria (MAU) and advanced glycation end products (AGEs) levels in diabetic and hypertensive subjects. Indian J Clin Biochem.

[15] Hartmann D, Thum T (2011). MicroRNAs and vascular (dys)function. Vasc Pharmacol.

[16] He F-F, Chen S, Su H, Meng X-F, Zhang C (2013). Actin-associated proteins in the pathogenesis of podocyte injury. Curr Genomics.

[17] Heeg JE, de Jong PE, van der Hem GK, de Zeeuw D (1987). Reduction of proteinuria by angiotensin converting enzyme inhibition. Kidney Int.

[18] Hirao Y, Saito S, Fujinaka H, Miyazaki S, Xu B, Quadery AF (2018). Proteome profiling of diabetic mellitus patient urine for discovery of biomarkers by comprehensive ms-based proteomics. Proteomes.

[19] Hovind P, Rossing P, Tarnow L, Smidt UM, Parving H-H (2001). Progression of diabetic nephropathy. Kidney Int.

[20] Huminiecki L (2019). Magic roundabout is an endothelial-specific ohnolog of ROBO1 which neo-functionalized to an essential new role in angiogenesis. PLoS One.

[21] Ikeda S, Makino H, Haramoto T, Shikata K, Kumagai I, Ota Z (1991). Changes in glomerular extracellular matrices components in diabetic nephropathy. J Diabetes Complicat.

[22] Jefferson JA, Shankland SJ, Pichler RH (2008). Proteinuria in diabetic kidney disease: A mechanistic viewpoint. Kidney Int.

[23] Jin J, Ku YH, Kim Y, Kim Y, Kim K, Lee JY (2012). Differential proteome profiling using iTRAQ in microalbuminuric and normoalbuminuric type 2 diabetic patients. Exp Diabetes Res.

[24] Katsuki A, Sumida Y, Murashima S, Murata K, Takarada Y, Ito K (1998). Serum levels of tumor necrosis factor-alpha are increased in obese patients with noninsulin-dependent diabetes mellitus. J Clin Endocrinol Metab.

[25] Lee SY, Choi ME (2015). Urinary biomarkers for early diabetic nephropathy: beyond albuminuria. Pediatr Nephrol.

[26] Lewandowicz A, Bakun M, Kohutnicki R, Fabijanska A, Kistowski M, Imiela J (2015). Changes in urine proteome accompanying diabetic nephropathy progression. Pol Arch Med Wewn.

[27] Li Q, Ye Z, Wen J, Ma L, He Y, Lian G (2009). Gelsolin, but not its cleavage, is required for TNF-induced ROS generation and apoptosis in MCF-7 cells. Biochem Biophys Res Commun.

[28] Lin L, Yu Q, Zheng J, Cai Z, Tian R (2018). Fast quantitative urinary proteomic profiling workflow for biomarker discovery in kidney cancer. Clin Proteom.

[29] Lu H, Deng S, Zheng M, Hu K (2019). iTRAQ plasma proteomics analysis for candidate biomarkers of type 2 incipient diabetic nephropathy. Clin Proteom.

[30] Ludwig C, Gillet L, Rosenberger G, Amon S, Collins BC, Aebersold R (2018). Data-independent acquisition-based SWATH-MS for quantitative proteomics: a tutorial. Mol Syst Biol.

[31] Mackinnon B, Shakerdi L, Deighan CJ, Fox JG, O'Reilly DS, Boulton-Jones M (2003). Urinary transferrin, high molecular weight proteinuria and the progression of renal disease. Clin Nephrol.

[32] Marimuthu A, O'Meally RN, Chaerkady R, Subbannayya Y, Nanjappa V, Kumar P (2011). A comprehensive map of the human urinary proteome. J Proteome Res.

[33] Merchant ML, Niewczas MA, Ficociello LH, Lukenbill JA, Wilkey DW, Li M (2013). Plasma kininogen and kininogen fragments are biomarkers of progressive renal decline in type 1 diabetes. Kidney Int.

[34] Merchant ML, Perkins BA, Boratyn GM, Ficociello LH, Wilkey DW, Barati MT (2009). Urinary peptidome may predict renal function decline in type 1 diabetes and microalbuminuria. J Am Soc Nephrol.

[35] Muntel J, Xuan Y, Berger ST, Reiter L, Bachur R, Kentsis A (2015). Advancing urinary protein biomarker discovery by data-independent acquisition on a quadrupole-orbitrap mass spectrometer. J Proteome Res.

[36] Narita T, Sasaki H, Hosoba M, Miura T, Yoshioka N, Morii T (2004). Parallel increase in urinary excretion rates of immunoglobulin G, ceruloplasmin, transferrin, and orosomucoid in normoalbuminuric type 2 diabetic patients. Diabetes Care.

[37] Onile OS, Calder B, Soares NC, Anumudu CI, Blackburn JM (2017). Quantitative label-free proteomic analysis of human urine to identify novel candidate protein biomarkers for schistosomiasis. PLoS Negl Trop Dis.

[38] Overgaard AJ, Thingholm TE, Larsen MR, Tarnow L, Rossing P, McGuire JN (2010). Quantitative iTRAQ-based proteomic identification of candidate biomarkers for diabetic nephropathy in plasma of type 1 diabetic patients. Clin Proteom.

[39] Palm F, Cederberg J, Hansell P, Liss P, Carlsson PO (2003). Reactive oxygen species cause diabetes-induced decrease in renal oxygen tension. Diabetologia.

[40] Papale M, Di Paolo S, Magistroni R, Lamacchia O, Di Palma AM, De Mattia A (2010). Urine proteome analysis may allow noninvasive differential diagnosis of diabetic nephropathy. Diabetes Care.

[41] Pastushkova LK, Kashirina DN, Kononikhin AS, Brzhozovsky AG, Ivanisenko VA, Tiys ES (2018). The effect of long-term space flights on human urine proteins functionally related to endothelium. Hum Physiol.

[42] Pavkov ME, Knowler WC, Bennett PH, Looker HC, Krakoff J, Nelson RG (2006). Increasing incidence of proteinuria and declining incidence of end-stage renal disease in diabetic Pima Indians. Kidney Int.

[43] Perkins BA, Ficociello LH, Ostrander BE, Silva KH, Weinberg J, Warram JH (2007). Microalbuminuria and the risk for early progressive renal function decline in type 1 diabetes. J Am Soc Nephrol.

[44] Pickup JC, Chusney GD, Thomas SM, Burt D (2000). Plasma interleukin-6, tumour necrosis factor alpha and blood cytokine production in type 2 diabetes. Life Sci.

[45] Pieper R, Gatlin CL, McGrath AM, Makusky AJ, Mondal M, Seonarain M (2004). Characterization of the human urinary proteome: a method for high-resolution display of urinary proteins on two-dimensional electrophoresis gels with a yield of nearly 1400 distinct protein spots. Proteomics.

[46] Rawat P, Bathla S, Baithalu R, Yadav ML, Kumar S, Ali SA (2016). Identification of potential protein biomarkers for early detection of pregnancy in cow urine using 2D DIGE and label free quantitation. Clin Proteom.

[47] Sabapathy V, Stremska ME, Mohammad S, Corey RL, Sharma PR, Sharma R (2019). Novel Immunomodulatory cytokine regulates inflammation, diabetes, and obesity to protect from diabetic nephropathy. Front Pharmacol.

[48] Sagawa N, Fujita H, Banno Y, Nozawa Y, Katoh H, Kuzumaki N (2003). Gelsolin suppresses tumorigenicity through inhibiting PKC activation in a human lung cancer cell line, PC10. Br J Cancer.

[49] Sagoo MK, Gnudi L (2018). Diabetic nephropathy: Is there a role for oxidative stress?. Free Radic Biol Med.

[50] Shoukry A, Bdeer Sel A, El-Sokkary RH (2015). Urinary monocyte chemoattractant protein-1 and vitamin D-binding protein as biomarkers for early detection of diabetic nephropathy in type 2 diabetes mellitus. Mol Cell Biochem.

[51] Singh DK, Winocour P, Farrington K (2008). Mechanisms of disease: the hypoxic tubular hypothesis of diabetic nephropathy. Nat Clin Pract Nephrol.

[52] Sun J, Liu C (2018). Correlation of vascular endothelial function and coagulation factors with renal function and inflammatory factors in patients with diabetic nephropathy. Exp Ther Med.

[53] Tam FW, Riser BL, Meeran K, Rambow J, Pusey CD, Frankel AH (2009). Urinary monocyte chemoattractant protein-1 (MCP-1) and connective tissue growth factor (CCN2) as prognostic markers for progression of diabetic nephropathy. Cytokine.

[54] Tervaert TW, Mooyaart AL, Amann K, Cohen AH, Cook HT, Drachenberg CB (2010). Pathologic classification of diabetic nephropathy. J Am Soc Nephrol.

[55] Tomita H, Sanford RB, Smithies O, Kakoki M (2012). The kallikrein-kinin system in diabetic nephropathy. Kidney Int.

[56] Tziomalos K, Athyros VG (2015). Diabetic nephropathy: new risk factors and improvements in diagnosis. Rev Diabet Stud.

[57] Vitova L, Tuma Z, Moravec J, Kvapil M, Matejovic M, Mares J (2017). Early urinary biomarkers of diabetic nephropathy in type 1 diabetes mellitus show involvement of kallikrein-kinin system. BMC Nephrol.

[58] Wang Y, Chen J, Chen L, Zheng P, Xu HB, Lu J (2014). Urinary peptidomics identifies potential biomarkers for major depressive disorder. Psychiatry Res.

[59] Yagame M, Kim Y, Zhu D, Suzuki D, Eguchi K, Nomoto Y (1995). Differential distribution of type IV collagen chains in patients with diabetic nephropathy in non-insulin-dependent diabetes mellitus. Nephron.

[60] Yu Y, Sikorski P, Smith M, Bowman-Gholston C, Cacciabeve N, Nelson KE (2017). Comprehensive metaproteomic analyses of urine in the presence and absence of neutrophil-associated inflammation in the urinary tract. Theranostics.

[61] Zhang D, Ye S, Pan T (2019). The role of serum and urinary biomarkers in the diagnosis of early diabetic nephropathy in patients with type 2 diabetes. PeerJ.

[62] Zhu D, Kim Y, Steffes MW, Groppoli TJ, Butkowski RJ, Mauer SM (1994). Glomerular distribution of type IV collagen in diabetes by high resolution quantitative immunochemistry. Kidney Int.

